# The age-related expression patterns of *Larix kaempferi* *AP2* subfamily genes and functional dissection of *LkTOE1-2* in seed formation and germination

**DOI:** 10.48130/forres-0025-0028

**Published:** 2025-11-19

**Authors:** Zha-Long Ye, Xiang-Yi Li, Man-Li Nong, Xiao-Mei Sun, Wanfeng Li

**Affiliations:** 1 State Key Laboratory of Tree Genetics and Breeding, Key Laboratory of Tree Breeding and Cultivation of the National Forestry and Grassland Administration, Research Institute of Forestry, Chinese Academy of Forestry Beijing 100091, China; 2 Umeå Plant Science Centre (UPSC), Department of Plant Physiology, Umeå University 90187, Umeå, Sweden

**Keywords:** Aging, *APETALA2*, Gymnosperms, Phase transition, RUBY reporter system, Somatic embryogenesis

## Abstract

Conifers pose challenges for breeding programs due to their extended vegetative phases. Despite the critical role of *APETALA2* (*AP2*) in regulating phase transitions, the *AP2*/*ERF* superfamily remains largely unexplored in gymnosperms. Here, the first genome-wide analysis of the *AP2*/*ERF* superfamily in a conifer, *Larix kaempferi* (Japanese larch) is presented, and 374 members were identified. Among all eight paralogs, four *euAP2* lineage genes, *TARGET OF EATs* (*TOEs*), exhibit age-decreased expression patterns. Functional characterization of *LkTOE1-2* demonstrates its involvement in somatic embryogenesis and seed germination. Importantly, the RUBY reporter system confirmed an enhanced promoter activity in somatic embryo maturation. Over-expression of *LkTOE1-2* in *Arabidopsis* accelerates seed germination by 23.77%, bolting by 6.93%, and flowering by 5.92%. This study provides not only an expanded genomic resource for gymnosperms but also a candidate gene (*LkTOE1-2*) to shorten the juvenile phase of larch via molecular breeding.

## Introduction

As major coniferous timber species, larches (*Larix* spp.) hold substantial economic and ecological value due to their rapid growth and adaptability. However, their extended vegetative phase poses severe constraints for traditional breeding programs^[1,2]^. The plant life cycle progresses through distinct phases: dry seed, seed germination, juvenile and adult vegetative phase, followed by the reproductive phase, embryogenesis, seed maturation, and eventually seed set^[3]^. Thus, accelerating the life-cycle progression, especially the transition from the vegetative to the reproductive phases, is essential for improving genetic selection and promoting sustainable forestry.

In short-lived angiosperms such as *Arabidopsis thaliana*, phase transitions are precisely controlled by gene networks that integrate environmental and endogenous signals. Key regulators include the microRNA156 (miR156)-*SQUAMOSA PROMOTER BINDING PROTEIN-LIKE* (*SPL*) and miRNA172-*APETALA2* (*AP2*) modules^[4,5]^. miR172 represses *AP2*-like transcription factors to promote the vegetative phase transition, and reproductive phase transition, while stage-specific hormones further refine these processes^[6,7]^. While these pathways are well-studied in annual plants, their roles in conifers, which have long life cycles and distinct evolutionary histories, remain poorly understood. Understanding these mechanisms is significant for studying the genetic control of the development timing of coniferous trees.

The *AP2*/*Ethylene Responsive Factor* (*AP2*/*ERF*) transcription factor superfamily plays key roles in plant growth, phase transition, and stress responses^[8−10]^. This superfamily is classified into five major subfamilies—*AP2*, *dehydration response element-binding protein* (*DREB*), *ERF*, related to *ABA-insensitive3/viviparous1* (*RAV)*, and *Soloist*—based on the number and structure of their AP2 domains^[10]^. The *A. thaliana AP2* subfamily includes *AP2* and five *AP2*-like genes: *SCHLAFMUTZE* (*SMZ*), *SCHNARCHZAPFEN* (*SNZ*), *TARGET OF EAT1* (*TOE1*), *TOE2*, and *TOE3*, all of which contain two AP2 domains^[11]^. These genes are post-transcriptionally regulated by miR172 and function as repressors of the vegetative phase, and reproductive phase transition^[5,12−15]^. The *AP2* subfamily also includes *AINTEGUMENTA* (*ANT*) and *PLETHORA* (*PLT*), which are essential for floral organ identity and root growth, respectively^[16−18]^. *AP2* also regulates other life-cycle events, such as embryo development and seed germination^[19−21]^. The *DREB* and *ERF* subfamilies contain genes with a single AP2 domain. *DREB* genes play critical roles in abiotic stress responses, such as drought, cold, and salinity, while *ERF* genes regulate hormone signaling, pathogen defense, and abiotic stress tolerance^[10]^. The *RAV* subfamily, which contains both an AP2 domain and a B3 domain, influences the reproductive phase transition as well as biotic and abiotic stress responses^[22]^. The *Soloist* subfamily genes also contain a single AP2 domain, but their sequences and gene structures strongly diverge from those of the *DREB* and *ERF* subfamilies^[23]^.

In gymnosperms, functional studies of *AP2* superfamily genes remain limited. Recently, *Ginkgo biloba TOE1* is reported to enhance salt/drought tolerance, but its role in development is unknown^[24]^. In *G. biloba* and *Gnetum gnemon*, some *AP2* subfamily genes are broadly expressed during the early stages of ovule development and later are specifically expressed in the nucellus and integument, suggesting their involvement in embryogenesis and seed formation^[25]^. *Pinus thunbergii AP2-like 1*/*2* (*PtAP2L1*/*2*) are found to be expressed throughout cone development^[26]^, while *PaAP2L2* in *Picea abies* shows broad vegetative expression but remains functionally uncharacterized^[27]^. In *Larix kaempferi* (Japanese larch), *AP2/ERF* superfamily members such as *LkAP2L2* and *LkERF6* are found to be involved in branch formation, seed development, and stress responses^[28,29]^. Nevertheless, the functional roles of *AP2*/*ERF* superfamily genes in most gymnosperms—particularly *euAP2* lineage homologs—in regulating key life-cycle progression, remain largely unexplored. This gap limits our understanding of the distinctive longevity and reproductive strategies in gymnosperms. Some gymnosperm *AP2*/*ERF* superfamily genes contain a miR172 target site^[30]^, but their function in phase transition is unclear. Recent progress in conifer genomics^[31−35]^, including the sequencing of the *L. kaempferi* genome, now enables the use of molecular approaches to study phase transitions, and identify targets for genetic improvement through genome-wide studies of *L. kaempferi* *AP2*/*ERF* superfamily genes.

Here, genome-wide identification of the *AP2*/*ERF* superfamily genes in *L. kaempferi* were integrated with spatiotemporal expression profiling across several life-cycle stages, including seed germination, seedling development, vegetative and reproductive growth, embryogenesis, and seed maturation, to investigate their roles in life-cycle progression. Using RNA-seq, qRT-PCR, the RUBY reporter system, and transgenic *Arabidopsis*, a developmental stage specifically expressed *AP2* gene was characterized. The present findings will not only reveal an age-decreased *euAP2* lineage, but also provide a candidate gene for promoting seed formation and germination in larch breeding.

## Materials and methods

### Identification of the *AP2*/*ERF* superfamily genes in *L. kaempferi*

The genome files of *L. kaempferi* were obtained from the *Larix* Genome Database (LarixGD, www.Larixgd.cn/#/map)^[34]^. The Hidden Markov model (HMM) profiles of the AP2 domain (PF00847), and B3 domain (PF02362) were retrieved from the PFAM database (http://pfam.xfam.org). Genes containing these domains were scanned in the *L. kaempferi* genome using TBtools-HMMER with an *E*-value threshold of 10^−5[36]^.

To confirm these candidate genes, their predicted protein sequences were analyzed for conserved structural domains in the NCBI CDD database (www.ncbi.nlm.nih.gov/Structure/bwrpsb/bwrpsb.cgi), with an *E*-value cutoff of < 10^−5^. Genes were classified into subfamilies (*AP2, DREB, ERF, RAV,* and *Soloist*), based on domain type and number^[23]^.

### Protein physicochemical properties and subcellular localization prediction

The physicochemical properties, including aliphatic index, grand average of hydropathicity, instability index, molecular weight, and isoelectric point, were analyzed using TBtools software^[36]^. The potential subcellular localizations of these AP2/ERF superfamily proteins were predicted using WoLF PSORT (https://wolfpsort.hgc.jp).

### Multiple sequence alignment and phylogenetic analysis

To identify the *Soloist* subfamily genes, T-coffee scores of genes containing a single AP2 domain were obtained through multiple sequence alignment using MEGA12.0.9 software^[37]^. The genes with low scores were classified into the *Soloist* subfamily.

Atypical *ERF* and *DREB* genes in *L. kaempferi* were classified based on their evolutionary relationships. The protein sequences of typical *ERF* and *DREB* genes from *A. thaliana* were obtained from the *A. thaliana* information resource TAIR (www.arabidopsis.org/). Firstly, protein sequences of *A. thaliana* and *L. kaempferi* genes were aligned and trimmed using trimAI software^[38]^. Next, these sequences were used to construct phylogenetic trees in MEGA12.0.9 software^[37]^ using the maximum likelihood method (1,000 bootstraps), and the results were visualized in Evolview (https://evolgenius.info//evolview-v2).

For phylogenetic analysis of *AP2* subfamily genes in *A. thaliana* and *L. kaempferi*, a maximum likelihood tree was constructed with the same method as above. To predict miR172 target sites, the coding sequences of *AP2* subfamily genes were aligned with *A. thaliana AP2*, and the results were visualized with JalView software^[39]^.

### Analysis of the expression patterns of *AP2* subfamily genes using the age-related transcriptomes

To investigate the expression patterns of *AP2* subfamily genes during *L. kaempferi* tree aging, transcriptome data were obtained from the NCBI sequence read archive under BioProject accession: PRJNA234461. Branches from the upper crowns collected from dormant trees at ages 1, 4, 8, 12, 20, and 50 years^[40]^, and the uppermost main stems collected from active trees at ages 1, 2, 5, 10, 25, and 50 years were used for transcriptome sequencing^[41]^. After the removal of buds or needles, the left stems from at least three trees from each category were pooled and used for RNA extraction. These clean reads were mapped to the *L. kaempferi* genome^[34]^, and gene expression levels were calculated as transcripts per million (TPM). Heatmaps were generated in TBtools software, using the normalized TPM (log_2_) values.

### Plant materials used for qRT-PCR analysis

#### Experiment I: expression patterns of LkTOE1-2 during L. kaempferi tree aging

Samples from 0.58-, 2.58-, 4.5-, 5.58-, 8.58-, 10.50-, and 12.50-year-old dormant *L. kaempferi* trees were collected on 11 November 2020, while samples from 1.25-, 3.17-, 5.17-, 7.25-, 9.17-, 11.17-, and 13.17-year-old active *L. kaempferi* trees were collected on 4 July 2019. These samples were collected in our previous study^[42]^. The exact growth time was calculated based on afforestation records from the Dagujia seed orchard (42°22' N, 124°51' E), Liaoning Province, Northeast China. For 0.58-year-old trees, the main stem was sampled, while for all other age groups, lateral branches from the uppermost main stem, formed during the current year, were used. Buds and needles were removed before pooling samples from at least three trees per age group. Samples were then frozen in liquid nitrogen and stored at –80 °C until RNA extraction.

#### Experiment II and III: expression patterns of LkTOE1-2 in L. kaempferi seeds and seedlings

Mature seeds were collected from 38-year-old trees on 22 August 2020. Endosperm and embryo samples were taken from ten mature seeds after two days of water soaking. Ten 22-day-old seedlings were sampled on 13 May 2021, while ten 54-day-old, and six 74-day-old seedlings were sampled on 14 June and 4 July 2021, respectively. To analyze the expression pattern of *LkTOE1-2* in different organs, roots, hypocotyls, stems, and needles were collected from 15 74-day-old seedlings. All seedlings were sown on 22 April 2021. Before sowing, seeds were soaked in water until the embryonic root emerged through the seed coat. These samples were collected in our previous study^[42]^.

#### Experiment IV: expression patterns of LkTOE1-2 during L. kaempferi seed formation

Pollen grains were collected from 20 microsporophylls of 13-year-old trees on 13 April 2020. Immature embryos, endosperms, and seed coats were collected from ten immature seed cones on 30 June and 27 July 2022. Mature embryos, endosperms, and seed coats were collected from ten mature seed cones on 22 August 2020. These samples were collected in our previous study^[42]^.

#### Experiment V: expression patterns of LkTOE1-2 during L. kaempferi somatic embryogenesis

Callus was induced from immature embryos of *L. kaempferi* with the same medium as described in a previous study^[43]^. Other mediums used in somatic embryogenesis were according to the method of Song et al.^[44]^. Callus proliferation was maintained on proliferation medium (BM basal medium supplemented with 0.05 mg/L 6-BA, 0.05 mg/L KT, 0.50 mg/L NAA, 1 g/L glutamine, 0.50 g/L acid hydrolyzed casein, 25 g/L sucrose, 4 g/L agar, pH 5.75–5.85), under dark conditions, with subculturing every two weeks. Somatic embryos were induced from callus on maturation medium (BM basal medium with 15.86 mg/L ABA, 0.20 mg/L IBA, 10 g/L activated carbon, 68.40 g/L sucrose, 4 g/L agar, pH 5.75–5.85) for six weeks in the dark. Somatic seedlings were obtained by putting somatic embryos on germination medium (WPM basal medium containing 3 mg/L VB1, 2 g/L activated carbon, 20 g/L sucrose, 4 g/L agar, pH 5.75–5.85). The germination process consisted of a two-week dark culture followed by a one-month light culture.

#### Experiment VI: response of LkTOE1-2 to temperature treatment and water soaking

Seed cones were collected on 22 August 2020 and kept at room temperature for seed extraction. Group 1 was collected on 18 September 2020 from seeds that had not undergone 4 °C treatment, but were exposed to room temperature and water soaking. Groups 2–5 were collected from seeds stored at 4 °C for over a year. Group 2 was collected from seeds with no room temperature exposure or water soaking. Group 3 was collected from seeds with water soaking for two days without room temperature exposure. Group 4 was collected from seeds kept at room temperature for two weeks without water soaking. Group 5 was collected from seeds kept at room temperature for two weeks and with water soaking for two days. During water soaking, seeds were maintained at room temperature. For sampling, seed coats were removed from ten seeds and the remainder were collected.

### RNA extraction and qRT-PCR

Total RNA was extracted using the EasyPure RNA Kit (TransGen Biotech, ER101-01, Beijing, China) according to the manufacturer's instructions. A 2.5 µg aliquot of total RNA was reverse-transcribed into cDNA using the TransScript II One-step gDNA Removal and cDNA Synthesis SuperMix Kit (TransGen Biotech, Beijing, China), and then diluted for sequence cloning and qRT-PCR.

*LkTOE1-2* (also known as *LaAP2-1*, GenBank accession number: MN790757) was cloned in our previous work^[45]^. The primers 5'-GCGGACCAACAACTCCAGTA-3' and 5'-GTTGCCATATGCAAGCTCGG-3' were designed to amplify *LkTOE1-2*, 5'-GACTGTACCGTTGGTCGTG-3' and 5'-CCTCCAGCAGAGCTTCAT-3' for *L. kaempferi* *translation elongation factor-1 alpha 1* (*LkEF1A1*, GenBank Accession No.: JX157845), 5'-TTCGGCTTTGAAGGAGGGTC-3' and 5'-TTGGACGCAGTCCCCATAAG-3' for *L. kaempferi zinc finger protein* (*LkZFP*, GenBank Accession No.: MZ965074), 5'-TGGCGTCCAAAAGGATTCTCA-3' and 5'-TCCCATGATTGTAGCTTGCCA-3' for *L. kaempferi ubiquitin-conjugating enzyme E2 28* (*LaUBC1*, GenBank Accession No.: ON887160)^[42]^. *LkEF1A1* was used in all experiment, *LkZFP* was used in experiments I, and *LkUBC1* was used in experiments II–IV, and VI (Supplementary Table S1). ^△^Ct value (Ct_*LkEF1A*_ − Ct_*LkTOE1-2*_) was used for relative quantification analysis, where Ct represents the threshold cycle. Transcript levels shown in all figures are presented based on these values. QRT-PCR was performed with three or four technical replicates, and data were shown as mean ± SD. Statistical significance was assessed by one-way ANOVA followed by Tukey's multiple comparisons test.

### Expression patterns of *LkTOE1-2* during seed formation detected by RNA-seq

Immature embryos, endosperms, and seed coats were collected from ten immature seed cones on 9 July 2024. Mature seeds were collected from ten mature seed cones on 22 August 2020. All samples were frozen in liquid nitrogen, and then stored at −80 °C for RNA isolation. Transcriptome sequencing was performed on the Illumina HiSeqTM 2500 platform (Illumina, CA, USA) as described by Xiang et al.^[46]^. The raw reads have been uploaded to the China National Center for Bioinformation under Accession No.: PRJCA030694. Gene expression levels were calculated as above, and presented as means, with error bars representing the standard deviations (*n* = 3).

### Promoter cloning

The genomic DNA from *L. kaempferi* seedlings was extracted with the Plant Genomic DNA Kit (Tiangen Biotech, DP305-03, Beijing, China), according to the manufacturer's protocol. The sequences 2,000 bp upstream of *LkTOE1-2* ATG initiation codon were amplified using primers 5'-GTTAGCTCCGCGTTAGTTTTGAC-3', and 5'-CTTCGCTGCCGGAAGGCCAAGG-3'. PCR products were purified using a gel extraction kit (Tiangen, DP209-03, Beijing, China), and subsequently sequenced.

### Construction of *proLkTOE1-2::RUBY* vector and *L. kaempferi* transformation

The *proDR5::RUBY* vector was provided by Yubing He from the Institute of Crop Sciences, Chinese Academy of Agricultural Sciences. The promoter sequences of *LkTOE1-2* (*proLkTOE1-2*), and *CaMV35S* were separately subcloned into *proDR5::RUBY* vector via the HindIII cleavage site, replacing the *DR5* promoter^[47]^. This resulted in the *proLkTOE1-2::RUBY* and *pro35S::RUBY* vectors, respectively, with *proMV35S::RUBY* serving as a control. The primers 5'-gactgaccacccggggatccGTTAGCTCCGCGTTAGTTTTGAC-3', and 5'-gcgagggtcgcatgatccatCTTCGCTGCCGGAAGGCCAAGG-3' were used for *proLkTOE1-2*. The primers 5'-gactgaccacccggggatccTGAGACTTTTCAACAAAGGG-3' and 5'-gcgctgaagcttggctgcagTGTTCTCTCCAAATGAAATG-3' were used for *pro35S*. The recombinant vectors were first introduced into *Agrobacterium tumefaciens* strain GV3101, and subsequently transferred into *L. kaempferi* callus using a previously described method^[43]^.

### Construction of *pro35S::LkTOE1-2* vector and *A. thaliana* transformation

The coding sequence of *LkTOE1-2* was subcloned into the plant expression vector pBI121 via the BamHI cleavage site, and it was downstream of the *35S* promoter, resulting in the *pro35S::LkTOE1-2* vector. The primers 5'-tctagaaagcttCTGCAGATGATGACCAGAGATACTTCTCG-3', and 5'-ggtaccggatccACTAGTCGTGTCCCTTTGGACGCTTG-3' were used. The empty vector was used as a control. The recombinant vector was first introduced into *A. tumefaciens* strain GV3101, and subsequently transferred into *A. thaliana* ecotype Col-0 with the floral-dip method^[48]^. T1 transformants were selected on 1/2 Murashige and Skoog (MS) culture medium supplemented with 50 mg/L hygromycin. T2 transgenic plants were validated by PCR, RT-PCR, and qRT-PCR before phenotypic analysis (Supplementary Fig. S1). Plant growth conditions and statistical standards were the same as reported in our previous study^[49]^.

## Results and discussion

### Genome-wide identification of *AP2*/*ERF* genes and age-related expression patterns of *AP2* subfamily genes in *L. kaempferi*

In total, 374 genes were identified in the *L. kaempferi* genome, each containing at least one AP2 domain (Supplementary Table S2, Supplemental Figs S2, S3). These genes were assigned to five subfamilies, including eight *AP2*, 163 *DREB*, 158 *ERF*, 12 *RAV*, one *Soloist,* and 32 other subfamily genes (Table 1). Compared with *A. thaliana* (224 members), and *Oryza sativa* (178 members), the *AP2* subfamily in *L. kaempferi* is seven times fewer than in *A. thaliana* (56), and four times fewer than in *O. sativa* (32) (Table 1). The *DREB* subfamily in *L. kaempferi* expands significantly, because *L. kaempferi* has more members (163) than *A. thaliana* (eight), and *O. sativa* (five) (Table 1). This expansion may be linked to the complex genome evolution of conifers, including frequent whole-genome duplication events and adaptations to abiotic stresses such as cold, drought, and salinity^[31,33]^.

**Table 1:** Summary of the *AP2*/*ERF* superfamily in *Arabidopsis thaliana*, *Oryza sativa* and *Larix kaempferi.*

Classification	*Arabidopsis thaliana*	*Oryza sativa*	*Larix kaempferi*
*AP2* subfamily	56	32	8
*DREB* subfamily	8	5	163
*ERF* subfamily	134	127	158
Other subfamily	no	no	32
*RAV* subfamily	26	14	12
Soloist subfamily	Uncharacterized	Uncharacterized	1
Total	224	178	374
The data for *A. thaliana* and *O. sativa* *AP2*/*ERF* superfamily was from Choudhury^[63]^.			

**Figure 1:**
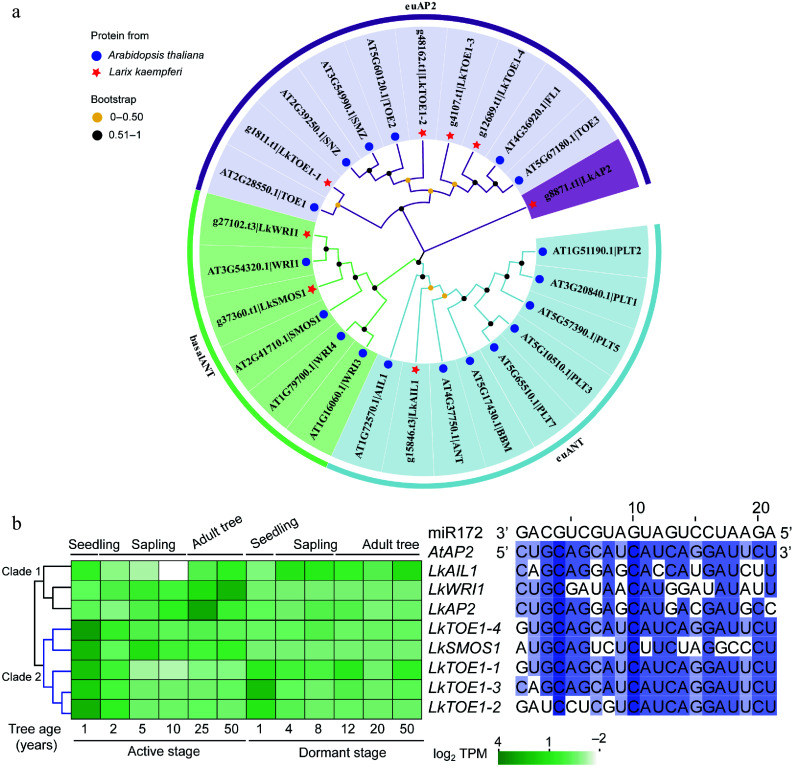
Phylogeny, sequence, and expression analysis of *AP2* subfamily genes in *Larix kaempferi*. (a) Phylogenetic tree of *AP2* subfamily proteins from *L. kaempferi* and *Arabidopsis thaliana*. (b) Sequence alignment of conserved miR172 target sites in *A. thaliana* and *L. kaempferi* *AP2* with *A. thaliana* miR172 as a reference and expression patterns of *AP2* subfamily genes during *L. kaempferi* tree aging detected by RNA-seq. Heatmaps were shown with the average normalized TPM (log_2_) values (–2 to 4).

Among the eight *AP2* subfamily members, *LkAP2* and *LkTOE1-1*/*2*/*3*/*4* belonged to the *euAP2* lineage, and *LkAIL1*, *LkSMOS1* and *LkWRI1* belonged to *ANT* lineage (Fig. 1a). The miR172 complementary sequences were present in *LkTOE1-1*/*2*/*3*/*4* (Fig. 1b)*,* suggesting their potential post-transcriptional regulation by miR172. *LkWRI1* and *LkAP2* showed increased expression with age in the active stage, whereas *LkTOE1-2* and *LkTOE1-3* displayed high expression in one-year-old seedlings followed by rapid decline in both the active and dormant stages (Fig. 1b). The 'early high, later silent' pattern of *LkTOE1-2*/*3* is similar to *TOE* homologs in *Arabidopsis*^[12]^, maize^[50]^, and *Cardamine flexuosa*^[51]^, *Pinus elliottii × P. caribaea*^[52]^ and other species^[53−55]^, suggesting a conserved regulatory role during early seedling development. Given that *LkTOE1-3* (also known as *LkAP2L2*) has been functionally characterized previously^[28]^, *LkTOE1-2* was selected for further investigation.

### *LkTOE1-2* transcription is regulated during seed formation and germination

**Figure 2:**
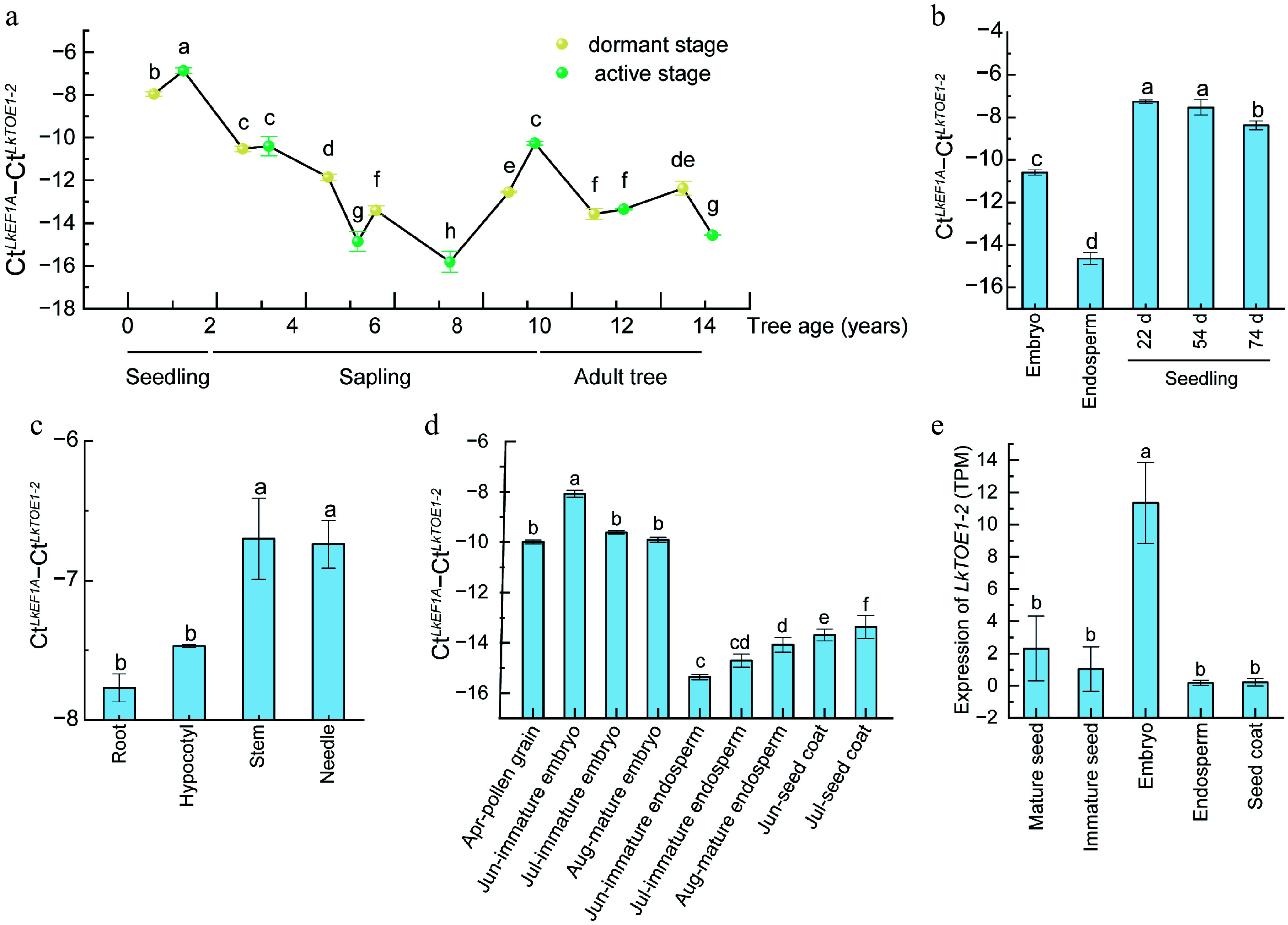
Expression patterns of *LkTOE1-2* during tree aging and seed formation in *Larix kaempferi*. (a) Expression patterns of *LkTOE1-2* during *L. kaempferi* tree aging detected by qRT-PCR. (b) Expression patterns of *LkTOE1-2* in seeds and seedlings. (c) Expression patterns of *LkTOE1-2* in different organs of seedlings. Expression patterns of *LkTOE1-2* during *L. kaempferi* seed formation detected by (d) qRT-PCR, and (e) RNA-seq. Different letters on the bars indicate significant differences as determined by one-way ANOVA followed by Tukey's multiple comparisons test, *p* < 0.05.

*LkTOE1-2* expression level was high in the active and dormant seedlings (Fig. 2a), indicating that it plays roles in early seedling development. Compared with seeds, *LkTOE1-2* expression level was elevated in seedlings, particularly in stems and needles (Fig. 2b, c). During seed development from June to August, *LkTOE1-2* was strongly expressed in embryos but weakly expressed in endosperms and seed coats (Fig. 2d, e). These findings suggested that *LkTOE1-2* transcription is induced during germination and is regulated during seed formation. These similar expression patterns of *LkTOE1-2* homologs in zygotic embryogenesis are also found in *G. biloba* and *G. gnemon*^[25]^, suggesting that these *AP2* subfamily genes function in zygotic embryogenesis.

### *LkTOE1-2* transcription was induced during *L. kaempferi* somatic embryo formation and germination

**Figure 3:**
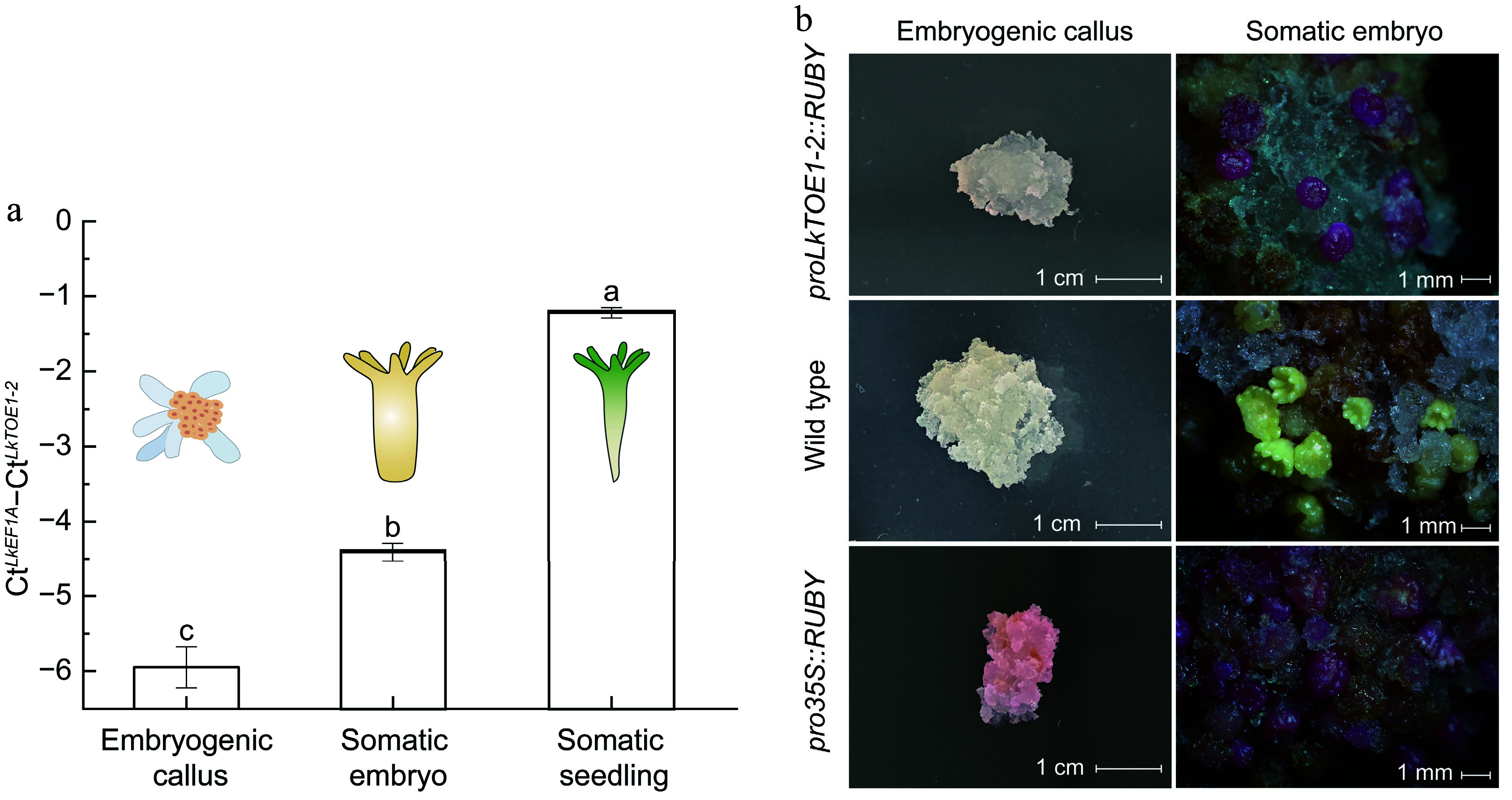
Expression patterns and promoter activity of *LkTOE1-2* during *Larix kaempferi* somatic embryogenesis. (a) Expression patterns of *LkTOE1-2* in embryogenetic calli, somatic embryos, and somatic seedlings detected by qRT-PCR. Different letters on the bars indicate significant differences as determined by one-way ANOVA followed by Tukey's multiple comparisons test, *p* < 0.05. (b) Promoter activity of *LkTOE1-2* in embryogenetic calli and somatic embryos detected with the RUBY reporter system.

During somatic embryogenesis, *LkTOE1-2* expression level was 1.54-fold higher in somatic embryos and 4.73-fold higher in somatic seedlings compared with embryogenic calli (Fig. 3a), showing that *LkTOE1-2* transcription is induced during *L. kaempferi* somatic embryo formation and germination. Using the RUBY reporter system^[47]^, enhanced promoter activity of *LkTOE1-2* was observed during embryo maturation. Wild-type calli appeared white, calli expressing *pro35S::RUBY* were red, and calli expressing *proTOE1-2::RUBY* were also white (Fig. 3b). After a maturation culture, light yellow somatic embryos formed from white wild-type calli, and red somatic embryos formed from red transgenic calli expressing *pro35S::RUBY* and white calli expressing *proTOE1-2::RUBY* (Fig. 3b). These visible observations showed that *LkTOE1-2* transcription is induced during somatic embryo formation. Unfortunately, these red transgenic somatic embryos did not develop into somatic seedlings. *L.* × *marschlinsii AP2L1* and *L. decidua BABYBOOM* (*BBM*) also have this similar expression pattern during somatic embryogenesis^[56,57]^, suggesting that these *AP2* subfamily genes function in somatic embryogenesis. Altogether, *AP2* subfamily genes function in both zygotic and somatic embryogenesis in gymnosperms, and are similar to *Arabidopsis* *BBM*^[58−60]^.

### *LkTOE1-2* transcription was induced by low temperature and following water soaking

**Figure 4:**
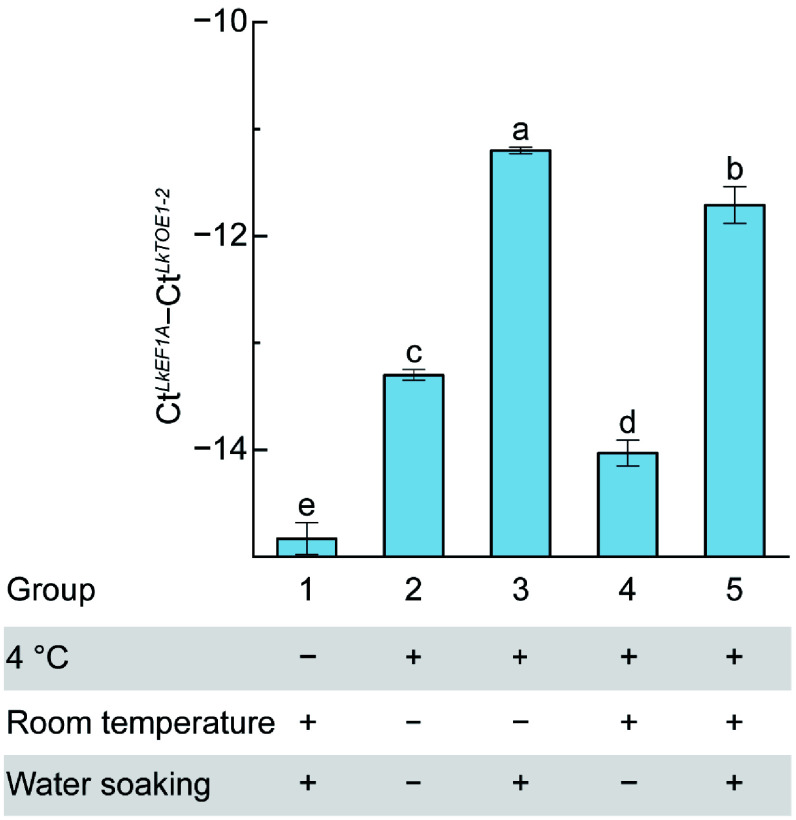
Responses of *LkTOE1-2* expression to temperature and water soaking detected by qRT-PCR. Different letters on the bars indicate significant differences as determined by one-way ANOVA followed by Tukey's multiple comparisons test, *p* < 0.05.

To investigate the regulatory mechanism of *LkTOE1-2* during seed germination, the impacts of two key factors essential for seed germination—low temperature and water soaking—on *LkTOE1-2* expression were examined. *LkTOE1-2* expression level was the lowest in group 1 that had not undergone 4 °C treatment (Fig. 4). It was high in groups 3 and 5 that had been treated with 4 °C and water soaking, and were low in groups 2 and 4 that had been treated with 4 °C, but without water soaking (Fig. 4). Compared with group 1, it was high in group 5, indicating that 4 °C treatment increased *LkTOE1-2* expression. Compared with group 2, it was high in group 3, and compared with group 4, it was high in group 5, indicating that water soaking increased *LkTOE1-2* expression (Fig. 4). Notably, compared with group 2 and 3, it did not increase in group 4 and 5, respectively, indicating that without water soaking, only room temperature treatment could not increase *LkTOE1-2* expression (Fig. 4). These data further showed that low temperature and the following water soaking induce *LkTOE1-2* transcription.

### *LkTOE1-2* over-expression promoted the germination, bolting, and the first flowering in *A. thaliana*

**Figure 5:**
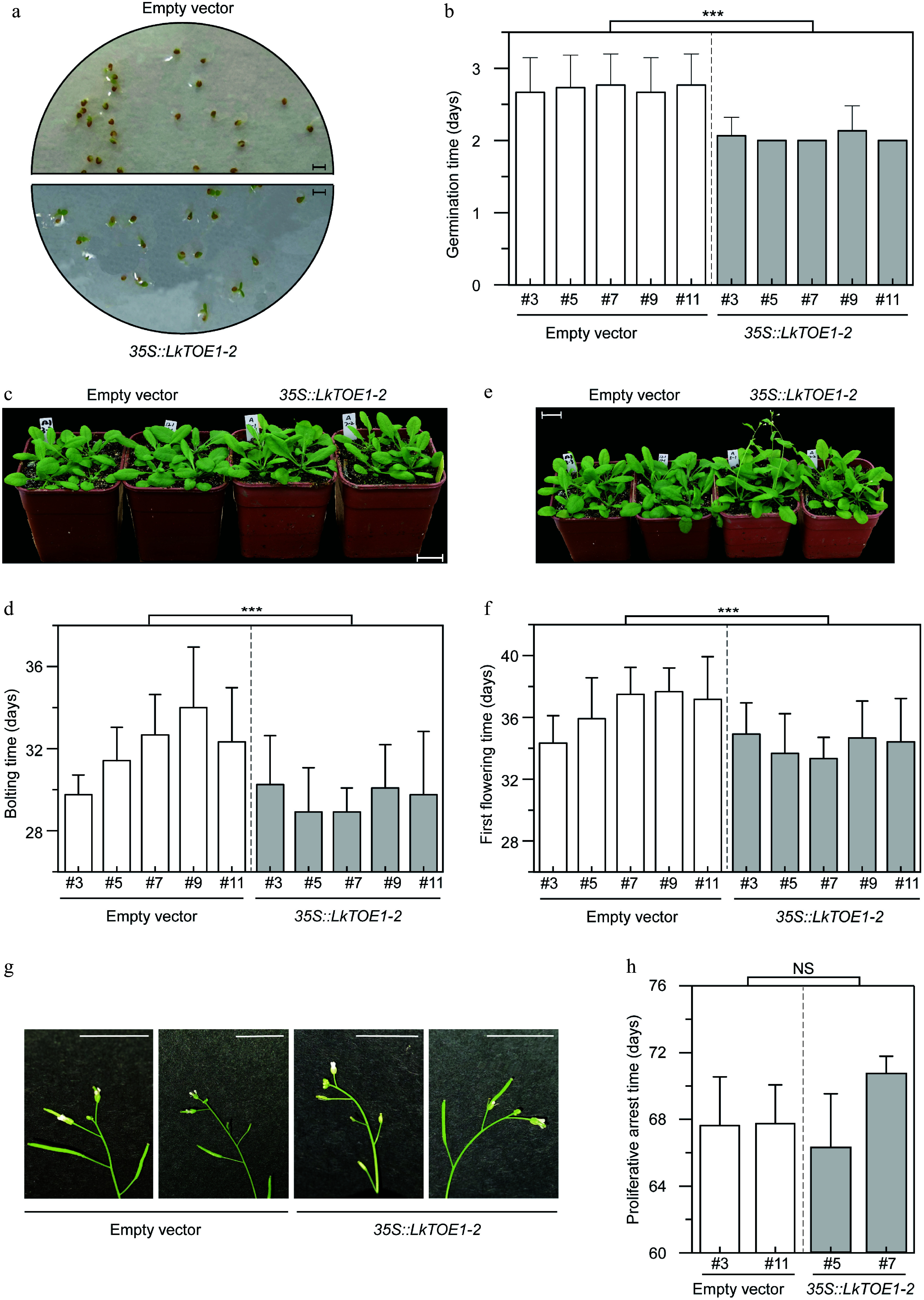
Phenotypes of transgenic *Arabidopsis thaliana* over-expressing *LkTOE1-2*. (a) Germination of transgenic *A. thaliana*. Scale bar = 3 mm. The pictures were taken on the second day after 4 °C treatment. (b) Germination time of transgenic *A. thaliana*. *n* = 30. Germination was counted when two cotyledons were visible. (c) Bolting of transgenic *A. thaliana*. Scale bar = 2 cm. The pictures were taken on the 29^th^ day after 4 °C treatment. (d) Bolting time of transgenic *A. thaliana*. Twelve plants were used in each line. Bolting was counted when inflorescence axis was visible, and bolting time was recorded from 4 °C treatment. *n* = 12. (e) Flowering of transgenic *A. thaliana*. Scale bar = 2 cm. The pictures were taken on the 31^th^ day after 4 °C treatment. (f) First flowering time of transgenic *A. thaliana* over-expressing *LkTOE1-2*. First flowering time was recorded from 4 °C treatment. *n* = 12. (g) Proliferative arrest of the inflorescence axis in transgenic *A. thaliana*. Scale bar = 1 cm. The pictures were taken on the 65^th^ day after 4 °C treatment. (h) Proliferative arrest time of the inflorescence axis in transgenic *A. thaliana*. Proliferative arrest was counted when a spherical structure is formed in the apex of the inflorescence axis, and proliferative arrest time was recorded from 4 °C treatment. *n* = 8. Transgenic *A. thaliana* expressing the empty vector was used as a control. *** *p* < 0.001, NS *p* ≥ 0.05, Student's *t*-test.

To test the function of *LkTOE1-2*, *LkTOE1-2* was over-expressed in *A. thaliana.* After measuring the germination of transgenic seeds, it was found that *LkTOE1-2* over-expression promoted the germination of *A. thaliana* seeds, as it took *A. thaliana* seeds over-expressing *LkTOE1-2* 2.02 d to germinate, while it took *A. thaliana* seeds expressing empty vector 2.65 d (Fig. 5a, b); it was ~0.63 d (23.77%) earlier (*p* < 0.001, Student' *t*-test). These data showed that *LkTOE1-2* possesses able to enhance seed germination.

To test the function of *LkTOE1-2* in the reproductive phase transition, the bolting time of transgenic *A. thaliana* was counted, and it was found that *LkTOE1-2* over-expression resulted in the early reproductive phase transition, because it took *A. thaliana* over-expressing *LkTOE1-2* 29.58 ± 2.26 d to bolt, while it took *A. thaliana* expressing empty vector 31.78 ± 2.42 d (Fig. 5c, d); it was ~2.20 d (6.93%) earlier (*p* < 0.001, Student' *t*-test).

It was also found that the first flowering time was promoted by *LkTOE1-2* over-expression, because it took *A. thaliana* over-expressing *LkTOE1-2* 34.17 ± 2.30 d and *A. thaliana* expressing empty vector 36.32 ± 2.54 d, respectively, to form the first flower (Fig. 5e, f); it was ~2.15 d (5.92%) earlier (*p* < 0.001, Student' *t*-test). The timing of proliferative arrest was not changed by *LkTOE1-2* over-expression (Fig. 5g, h, *p* = 0.38, Student' *t*-test).

Based on these data, it was concluded that the function of *LkTOE1-2* in bolting and flowering of *A. thaliana* was opposite to that of *AtAP2*^[5,12−15]^. The promotion of bolting (6.93%) and flowering (5.92%) was weaker than that of seed germination (23.77%), and it was speculated that this promotion might be affected by the early seed germination. Almost the same time was promoted in bolting and the first flowering, suggesting that the duration between bolting to the first flowering was not influenced by *LkTOE1-2* over-expression.

The functions of some gymnosperm homologs of *LkTOE1-2* in flowering have been revealed. In *P. abies*, *PaAP2L1* does not cause detectable changes in plant morphology or flowering time, whereas *PaAP2L2* delays flowering by an average of 4 d (19.0%) and increases the number of stamens and carpels in some flowers^[61]^. In *L. kaempferi*, *LkAP2L1* delays flowering by an average of 8.5 d (34.4%), and increases organ size, but has no effect on floral organ identity^[62]^; *LkAP2L2* increases shoot branching, although its effect on flowering time and floral organ identity is not prominent^[28]^. Collectively, these studies indicate that the functions of *AP2* subfamily genes in flowering are different, and the mechanism underlying this functional diversification needs further study.

In addition, the lifetime was not affected by *LkTOE1-2* because the proliferative arrest time is not changed in *A. thaliana* over-expressing *LkTOE1-2*. These results imply a potential role of *LkTOE1-2* for shortening the juvenile phase of larch via molecular breeding.

## Conclusions

This study provides the first comprehensive genome-wide identification and characterization of the *AP2/ERF* superfamily in *L. kaempferi*. Age-decreased expression patterns in the *euAP2* lineage genes (*LkTOE1-1/2/3/4*) were revealed. Functional dissection confirmed that *LkTOE1-2* expression is developmentally regulated during seed formation, somatic embryogenesis, and seed germination. Its promoter activity, validated by the RUBY reporter system, is enhanced during somatic embryo maturation. Over-expression of *LkTOE1-2* in *Arabidopsis* accelerates seed germination, bolting time, and flowering. Collectively, these findings highlight not only divergent regulatory pathways in *L. kaempferi* compared with angiosperms but also provide *LkTOE1-2* as a candidate gene for molecular breeding strategies aimed at shortening the juvenile phase and enhancing seed formation and germination in larch.

## SUPPLEMENTARY DATA

Supplementary data to this article can be found online.

## Data Availability

All data generated or analyzed during this study are included in the Supplemental Tables.
