# Supplementary figures and images for: The age-related expression patterns of Larix kaempferi AP2 subfamily genes and functional dissection of LkTOE1-2 in seed formation and germination
Source: For Res (Fayettev). 2025 Nov 19;5:e027. doi: 10.48130/forres-0025-0028 (PMC12648160; doi:10.48130/forres-0025-0028)

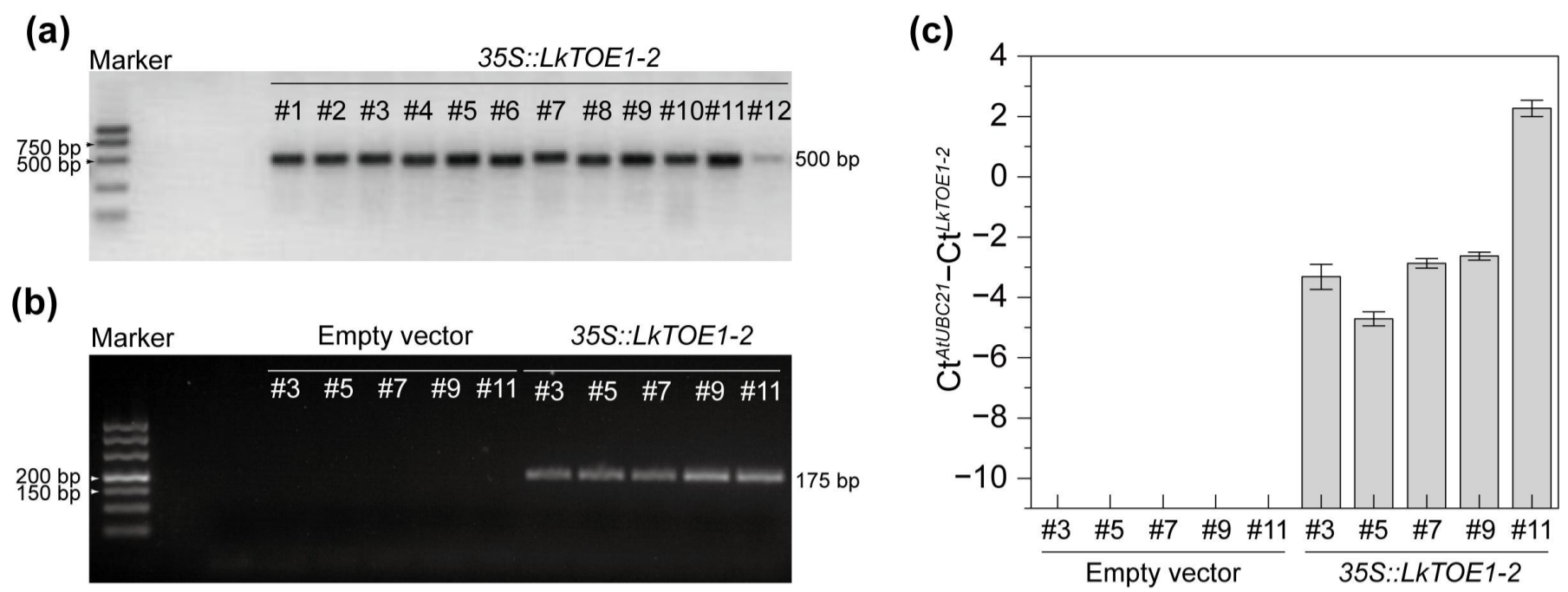

**Fig. S1** Verification of transgenic *Arabidopsis thaliana*.

Supplement: Supplementary file 1 — Supplementary data to this article can be found online. [file FR-2025-5-0028-Supplementary.zip › 10.48130_forres-0025-0028-Suppl-FigureS1.pdf]
