# Supplementary material for: The age-related expression patterns of Larix kaempferi AP2 subfamily genes and functional dissection of LkTOE1-2 in seed formation and germination
Source: For Res (Fayettev). 2025 Nov 19;5:e027. doi: 10.48130/forres-0025-0028 (PMC12648160; doi:10.48130/forres-0025-0028)
Supplement: Supplementary file 1 — Supplementary data to this article can be found online. [file 10.48130_forres-0025-0028-Suppl-FigureS3.pdf › 10.48130_forres-0025-0028-Suppl-FigureS3.pdf]

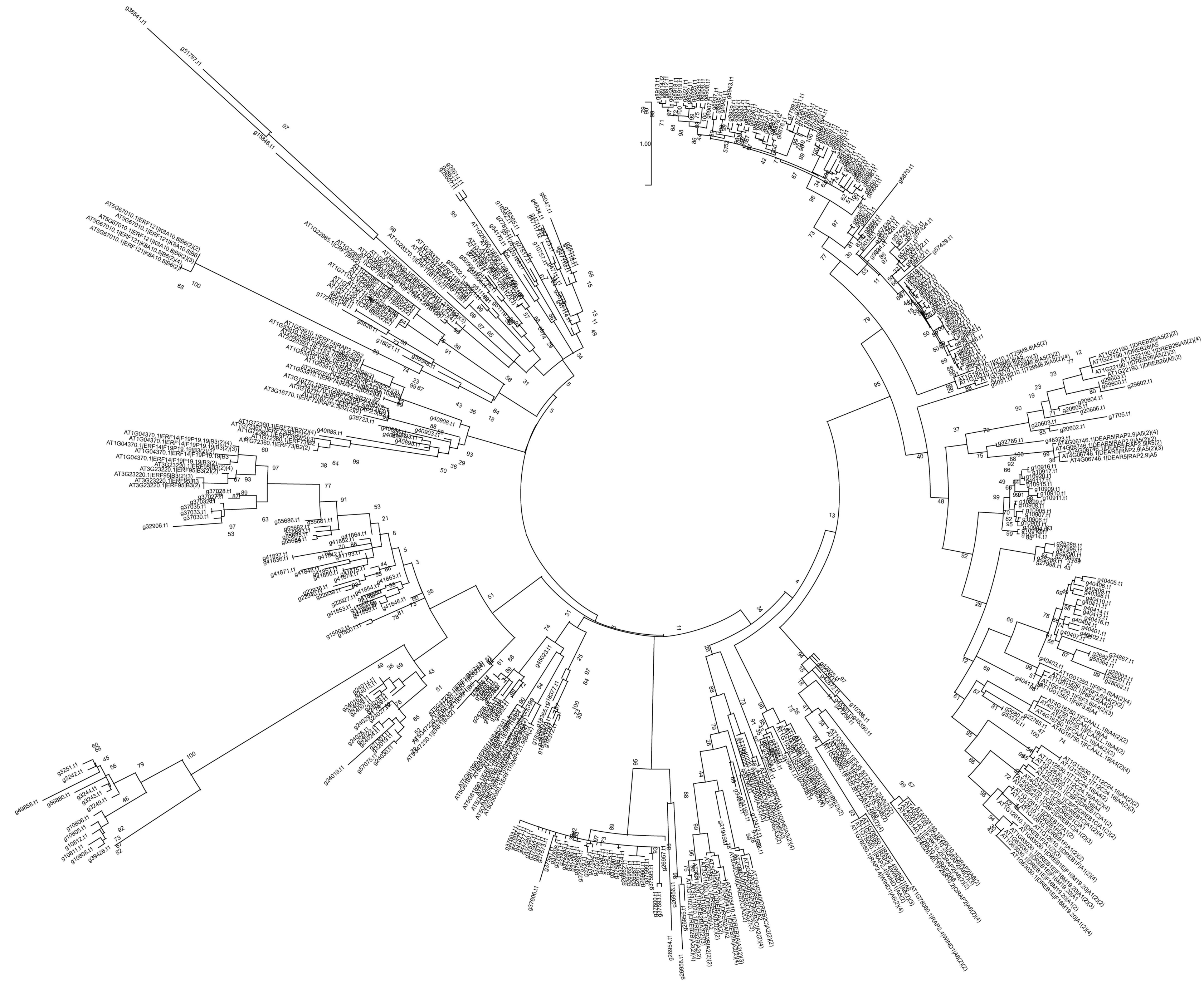

Fig. S3 Phylogenetic tree of AP2 proteins from *Arabidopsis thaliana* and *Larix kaempferi*, both containing a single AP2 domain.
